# The Development of a Pipeline for the Identification and Validation of Small-Molecule RelA Inhibitors for Use as Anti-Biofilm Drugs

**DOI:** 10.3390/microorganisms8091310

**Published:** 2020-08-28

**Authors:** Donald C. Hall, Jarosław E. Król, John P. Cahill, Hai-Feng Ji, Garth D. Ehrlich

**Affiliations:** 1 Department of Chemistry, Drexel University, Philadelphia, PA 19104, USA; dch66@drexel.edu (D.C.H.J.); jpc356@drexel.edu (J.P.C.); 2 Department of Microbiology & Immunology, Center for Advanced Microbial Processing, Drexel University, Philadelphia, PA 19102, USA; jek322@drexel.edu; 3 Center for Genomic Sciences, Drexel University, Philadelphia, PA 19102, USA; 4 Center for Surgical Infections and Bacterial Biofilms, Institute of Molecular Medicine, and Infectious Disease, Drexel University, Philadelphia, PA 19102, USA; 5 Department of Otolaryngology-Head and Neck Surgery, Drexel University College of Medicine, Drexel University, Philadelphia, PA 19102, USA

**Keywords:** biofilms, *in silico* docking, stringent response, persister cells, drug resistance, antibiotic support

## Abstract

Biofilm infections have no approved effective medical treatments and can only be disrupted via physical means. This means that any biofilm infection that is not addressable surgically can never be eliminated and can only be managed as a chronic disease. Therefore, there is an urgent need for the development of new classes of drugs that can target the metabolic mechanisms within biofilms which render them recalcitrant to traditional antibiotics. Persister cells within the biofilm structure may play a large role in the enhanced antibiotic recalcitrance of bacteria biofilms. Biofilm persister cells can be resistant to up to 1000 times the minimal inhibitory concentrations of many antibiotics, as compared to their planktonic envirovars; they are thought to be the prokaryotic equivalent of metazoan stem cells. Their metabolic resistance has been demonstrated to be an active process induced by the stringent response that is triggered by the ribosomally-associated enzyme RelA in response to amino acid starvation. This 84-kD pyrophosphokinase produces the “magic spot” alarmones, collectively called (p)ppGpp. These alarmones act by directly regulating transcription by binding to RNA polymerase. These transcriptional changes lead to a major shift in cellular function to both upregulate oxidative stress-combating enzymes and down regulate major cellular functions associated with growth and replication. These changes in gene expression produce the quiescent persister cells. In this work, we describe a hybrid *in silico* laboratory pipeline for identifying and validating small-molecule inhibitors of RelA for use in the combinatorial treatment of bacterial biofilms as re-potentiators of classical antibiotics.

## 1. Introduction

Biofilms can be defined as a multicellular stage in the bacterial life cycle wherein bacteria, through multiple intercellular communication systems, create densely populated communities embedded within a self-extruded extracellular polymeric matrix [1,2,3,4,5]. Biofilms can be resistant to antibiotic concentrations that are greater than 1000-times higher than their planktonic counterparts [6,7,8]; moreover, they also display the ability to live in extreme pHs [9]. These attributes make biofilm infections extremely difficult to eradicate and nearly impossible to eliminate if they are not accessible to physical means of disruption [3,4,5,10,11]. The activation of the ribosomally-associated RelA enzyme via amino acid starvation triggers the bacterial stringent response that leads to the phenotypic changes that underlie the extreme recalcitrance that biofilms exhibit towards antibiotics [12]. Thus, this ancient bacterial stress response produces an active metabolic state that results in the inability to treat chronic infections resulting from biofilms.

It has been known for more than half a century that the “magic spot” alarmones, guanine tetraphosphate and guanine pentaphosphate, collectively known as (p)ppGpp, produced by RelA, play an integral role in cell signaling for induction of the stringent response [13,14,15,16,17]. In *Escherichia coli*, the production of (p)ppGpp is carried out by the enzyme RelA [18]. RelA, which is a highly conserved 84-kD pyrophosphokinase protein among all eubacterial phyla [19], displays a well-choreographed dance with stalled ribosomes to detect amino acid starvation by means of deacetylated tRNAs [20]. Upon detection of this uncharged tRNA, RelA subsequently binds to the ribosomal complex and structurally changes to its active synthase conformation. While in this “open” conformation, RelA continually produces (p)ppGpp [21,22,23]. During this time, the intracellular concentrations of (p)ppGpp increase dramatically. The increased concentration of (p)ppGpp modulates multiple downstream cellular signaling pathways, including interacting with the RNA polymerase’s promoter binding region, thereby interfering with the cell’s ability to produce additional ribosomes [24].

Currently, there are only a very limited number of inhibitors known for RelA and (p)ppGpp that have been identified principally through traditional drug discovery methods, such as substrate analog design and high-throughput compound screening, none of which are candidates for clinical trials for the control of biofilm infections. The first of these inhibitors were analogs to ppGpp itself, such as Relacin and its derivatives [25]. These compounds, while mildly effective, suffer from off-target effects and low binding affinities [25,26,27,28,29].

The next compound discovered to reduce the intracellular concentrations of ppGpp was the cationic peptide known as IDR1018. This peptide is an analog to bactenecin [30,31,32,33] and it was reported to directly sequester and break down (p)ppGpp, thus lowering its intracellular concentration [30]. It is now thought that IDR1018 does not specifically target (p)ppGpp, but simply acts as an antimicrobial agent by means of its cationic nature [34]. Moreover, IDR1018 is a moderately sized peptide incapable of being an orally administered “druggable” compound.

Recently, a trend toward the use of *in silico* chemistry and molecular modeling for computer-aided drug design has gained significant momentum [35]. Previously, this was impossible to do with the RelA/RSH (RelA-SpoT homolog) family of enzymes, as there were no adequate high-resolution molecular structures available. However, several RelA and related enzyme structures have been recently characterized and published: RelA (*E. coli*) [36], RelP (*Staphylococcus aureus*) [37], RelQ (*S. aureus*) [38], Rel_seq_ (*Streptococcus equisimilis*) [39], and Rel (*Mycobacterium tuberculosis*) [40]. Thus, it has become possible through alignment and homology studies to determine the active residues within the catalytic center of these enzymes and to specifically target this region to predict and understand the ligand binding events for the rational identification of inhibitors.

## 2. Materials and Methods

### 2.1. Bacterial Strains and Growth Conditions

Bacterial strains are listed in Table S1. All bacterial strains were grown in Luria–Bertani broth (LB) or LB agar (1.5%). Antibiotics kanamycin (50 µg/mL), ampicillin (100 µg/mL), and chloramphenicol (25 µg/mL) were used when necessary. The druggable compounds S3-G1A and S3-G1B were purchased from Hit2Lead (ChemBridge Corporation, San Diego, CA, USA) and used at the concentrations described in the text.

### 2.2. Computational Docking

High-throughput *in silico* Docking Studies: The RelA enzyme (PDB: 5IQR) was prepared and optimized using Maestro Protein Preparation (Version 11.9.011, MMshare Version 4.5.011, Release 2019-1, Platform Windows-x64, Schrödinger Maestro, New York, NY, USA). The 5IQR PDB file contained extraneous portions of the ribosome, as the structure was determined as a RelA dimer associated with the ribosome. The ribosome and RNA subunits were removed and RelA was isolated in a separate file. The dockable RelA structure was prepared and minimized using Schrödinger’s protein preparation application [41]. This application was utilized to add hydrogens, create missing disulfide bonds, and determine lowest-energy residue orientations. Geometry minimization was carried out using the force field OPLS3e [42]. A docking site was determined using homology studies of bacterial *rel* genes from several species in combination with the Schrödinger binding site determination tool. Ligands were prepared using Schrödinger LigPrep (Schrödinger Release 2020-1: LigPrep, Schrödinger, LLC, New York, NY, USA, 2020). 

### 2.3. Biological Validation Assays

RelA Mutagenesis: The ASKA(-) clone JW2755-AM, containing an *E. coli* W3110 RelA in the pCA24N vector, was used for mutagenesis. A 1144-bp *Psi*I/*Nsi*I fragment was replaced by a synthetic construct. This construct contained 2 designated changes. First, a single nucleotide silent substitution (769 C/A) introduced at an *Xba*I restriction site, as described in [43,44]. Second, a TA/GC substitution at position 1027 replaced TAC (Y-310) with GCC (A-310). The 365-bp region between *Xba*I (769) and *Nsi*I (1144) contains the RelA active center and can be easily swapped with a synthetic construct to replace any of the tested amino acids. This method was applied to introduce the Y/A-319 mutation. A 365-bp *Xba*I/*Nsi*I fragment was replaced by a synthetic fragment with TAT-Y319 (position 1053) replaced with a GCC-A319 codon, as described in [45]. All mutations were confirmed by Sanger DNA sequencing.

RelA Protein Purification: The functional RelA enzyme and its Y/A-319 and Y/A-310 mutants were purified from host cell AG1 strains carrying the pJW2755-AM, pJEK2020-43, and pJEK2020-20 plasmids, respectively. One liter of LB broth was inoculated with 20 mL of overnight culture (OD_600_ = 0.9) and grown for 4 h (OD_600_ = 0.8) before induction with 1.5 mM IPTG for 4 h. Cultures were spun down, washed with phosphate-buffered saline (PBS), and resuspended in lysis buffer (50 mM NaH_2_PO_4_, 300 mM NaCl, 10 mM imidazole, pH 8.0) for lysing. To that resuspension, 1 µL/mL ThermoFisher Halt™ Protease Inhibitor Cocktail (100×) was added without EDTA and cells were lysed with sonication on ice (cycles of 10 s on and 10 s off for a total of 3 min of sonication, 2×). Lysates were spun down to remove cellular debris using a Sorvall RT7 Plus tabletop centrifuge (3.3 g, 15 min). Millipore Sigma PureProteome™ Nickel Magnetic Beads were used according to a modification of the manufacturer’s instructions. Supernatant was placed in 200 µL of nickel affinity beads for a period of 30 min. Beads were captured on a magnetic rack and the supernatant was removed. Beads were then washed 4× with wash buffer (50 mM NaH_2_PO_4_, 300 mM NaCl, 10 mM imidazole, pH 8.0). RelA was eluted twice using 300 mM imidazole elution buffer (50 mM NaH_2_PO_4_, 300 mM NaCl, 300 mM imidazole, pH 8.0) and a final elution using 500 mM imidazole elution buffer (50 mM NaH_2_PO_4_, 300 mM NaCl, 500 mM imidazole, pH 8.0). An SDS-page gel was run to confirm the presence and purity of RelA. Imidazole buffer was exchanged for PBS buffer and RelA was concentrated using Amicon^®^ Ultra-4 Centrifugal Filter Unit 30 kDa (MilliporeSigma, Burlington, MA, USA) nominal molecular weight limit. Nanodrop showed an average concentration of 1 mg/mL with a 260:280 ratio ~0.73.

Fluorescent Reporter RelA Activity Assay: The plasmid pAG001 (ampicillin 100 µg/mL), carrying a *yfp* fluorescent protein gene driven by the P*rpsJ* promoter, was used to detect the intracellular ppGpp concentrations, as published [46]. To validate the assay, this reporter plasmid, which is based on the broad host range RK2 minimal replicon, was introduced into *E. coli* K12 CF1648, and its *relA* mutants—(CF1652) [19], and AG1 (*relA1*) (NBRP Japan). To analyze the effect of overexpression of RelA and the Y/A310, and Y/A319 substitutions, ASKA plasmid pJW2755-AM [47] (chloramphenicol 25 µg/mL) and its derivatives—pJEK2020-20 with the Y/A310 mutation and pJEK2020-43 with the Y/A319 mutation—were extracted using the ThermoFisher Plasmid Mini DNA Extraction Kit, and transformed into AG1pAG001 strain (ampicillin 100 µg/mL, chloramphenicol 25 µg/mL). For the fluorescent RelA activity assay, overnight cultures of the selected strains were diluted 1:100 in fresh LB medium and 200 µL aliquots were placed into 96-well plates (Costar). The plates were placed in a Tecan Infinite M200 Pro Microplate Reader with a programmed growth cycle (18 h, 37 °C, orbital rotation 3.5). Cell density was measured at OD_600_ and YFP fluorescence activity was detected with 505 nm/535 nm (excitation/emission). Enzymatic activity was measured as Relative Fluorescence Units (RFU-YFP/OD_600_). 

In vitro (p)ppGpp quantification: In vitro (p)ppGpp quantification was carried out using techniques similar to those previously reported in the literature [25,26,28,48]. The RelA enzyme was purified, as described above. Roughly 0.4 µg of RelA protein was added to a 1.5 mL microcentrifuge tube containing a reaction mix composed of 1 × PBS, 5 mM MgCl_2_, 0.5 mM ATP, 0.5 mM GTP, 0.5 mM GDP, and 20 µCi[γ-32P]ATP (3000 Ci/mmol; PerkinElmer, Waltham, MA, USA) and varying concentrations of the compound of interest. These reactions were incubated at 37 °C for 1 h. The reactions were stopped by the addition of 5 µL formic acid (88%). The reaction mixtures were then spotted on a stationary-phase polyethyleneimine (PEI)-cellulose TLC plate (Sorbent Technologies, Norcross, GA, USA) using potassium phosphate monobasic (1.5 M) as the mobile-phase. The plates were then dried, and the radiation levels were read using a Molecular Dynamics Storage Phosphor Screen. A Molecular Dynamics Storm 840 Phosphor imager Scanner was used to read the phosphor screen and ImageJ was used to process the images. 

In vivo (p)ppGpp Quantification: In vivo (p)ppGpp quantification was carried out using techniques similar to those previously reported in the literature [25,26,28,48]. One milliliter of overnight cell culture of *E. coli* C was placed in 1.5 mL microcentrifuge tubes and pelleted. To this pellet, 50 µL of a reaction mixture containing 20 µCi orthophosphoric acid and 40 µM serine hydroxamate in 1× MOPS minimal medium was added. The cell pellet was resuspended by gentle vortexing and placed in an incubator for 1 h. Cell growth arrest and cell lysis were completed by the addition of 15 μL formic acid (88%). The lysate was then centrifuged to remove any insoluble components and the supernatant was spotted on a stationary-phase PEI-cellulose TLC plate. Plates were processed and analyzed as described above.

Biofilm dispersal assays: For biofilm formation on polystyrene surfaces, flat-bottom 96-well microtiter plates (Corning Inc., Corning, NY, USA) were used. Two hundred microliters of bacterial culture (100× diluted overnight culture; approximately 10^7^ cells) in fresh LB medium was added to each well. These were allowed to grow for 24 h. The planktonic cells and medium were then aspirated, and the plates were washed twice with 1× PBS, aspirating and discarding the PBS wash each time. Fresh LB with hit compounds was added to the biofilm wells. These cultures were then allowed to incubate at 37 °C overnight. Then, 100 µL of the planktonic culture was transferred to another 96-well plate without disturbing the underlying biofilm, and the cell density of the planktonic culture was measured (OD_600_) using a Multiscan Go plate reader (Thermo Fisher Scientific, Waltham, MA, USA). Biofilm volume was measured by adding 100 μL fresh LB and 30 μL Gram crystal violet (CV) (Remel, San Diego, CA, USA); 3 g crystal violet, 50 mL isopropanol, 50 mL ethanol, 900 mL purified water) to the original plate and allowing it to incubate for 1 h for staining. Plates were washed with water and air dried, and CV was solubilized with an ethanol:acetone (4:1) solution. The OD_570_ was determined from this solution, and the biofilm volume was calculated as the ratio of OD_570_ to OD_600_ [49,50].

Biofilm inhibition assays: For biofilm formation on polystyrene surfaces, flat-bottom 96-well microtiter plates (Corning Inc.) were used. The effect of different compounds on biofilm formation was tested by adding compounds at different concentrations to the bacterial culture (100× diluted overnight culture; approximately 10^7^ cells) in fresh LB medium. Two hundred microliter aliquots were pipetted into 96-well plates and placed for 24 or 48 h into a 37 °C incubator. The biofilm mass was measured by the CV staining assay described above.

Biofilm persistence assays with ampicillin: Biofilms were grown for 24 or 48 h as described above. Planktonic cells were removed, and the biofilms were washed twice with 250 µL sterile PBS solution. Two hundred microliters of fresh LB medium with various concentrations of ampicillin were dispensed into the wells. After 18 h of incubation at 37 °C, the volume of biofilm was measured by CV staining, as described above.

Synergistic effects of *in silico* ‘hit’ compounds and antibiotics: Biofilms were grown for 24, 48, or 72 h as described above. Planktonic cells were then removed, and biofilms were washed twice with 250 µL sterile PBS solution. Two hundred microliter aliquots of fresh LB medium with multiple concentrations of the ‘hit’ compounds to be tested and ampicillin were dispensed into the wells. After 18 h of incubation at 37 °C, the biofilm mass was measured as described above. For the alamarBlue viability test, 4 µL of alamarBlue (Invitrogen, Carlsbad, CA, USA) was added and plates were incubated in a BioTek HT plate reader at 37 °C for 4 h. Cell viability was measured as fluorescence at 530/590 nm (excitation/emission) versus compound concentration or initial cell density.

Cell growth curves: The effect of the hit compounds on bacterial growth was tested by adding compounds at multiple concentrations to the bacterial culture (100× diluted overnight culture; approximately 10^7^ cells) in fresh LB medium. Two hundred microliters of aliquots were pipetted into 96-well plates and placed into a BioTek HT (BioTek, Winooski, VT, USA) or Tecan Infinite M200 Pro (Tecan, Männedorf, Switzerland) plate reader for 18 h at 37 °C. Plates were shaken during incubation and the optical density (OD_630_ or OD_600_) was measured every 15 min.

Antibiotic susceptibility assays: For liquid cultures, the minimal inhibitory concentrations (MICs) of the antimicrobial drugs were determined using 96-well plates and the broth dilution method. Suspensions were then incubated at 37 °C for 18 h in a BioTek HT plate reader (see bacterial growth). Biofilm destruction experiments were performed with different antibiotic concentrations, and cell densities were measured after 18 h. Bacterial concentrations were calculated via optical density (OD_630_), and the lowest concentration causing 80% growth inhibition relative to the growth of the control was deemed to be the MIC.

Scanning electron microscopy (SEM) of biofilm: *E. coli* biofilms were grown in LB with multiple concentrations of the hit compounds on metal pins [51]. These metal pins were then washed twice in 1 × PBS. The biofilm-containing metal pins were then placed in a 5% glutaraldehyde solution for 1 h. Metal pins were then dehydrated using a gradient of ethanol from 50% to 100%—5 min in each solution. The pins were sputter coated with gold at a thickness of 60 Å. SEM images were taken on a Zeiss Supra 50VP Scanning Electron Microscope (Carl Zeiss AG, Oberkochen, Germany) with 5 kV beam acceleration.

Statistical analysis: Statistical analyses were performed using OriginPro 8.5 (Originlab Corporation, Northampton, MA, USA). Relevant statistical data are included in the results and discussion for each experiment. Error bars indicate standard deviation from the mean. Asterisks represent statistical significance of at least *p* < 0.05.

## 3. Results and Discussion

**Figure 1:**
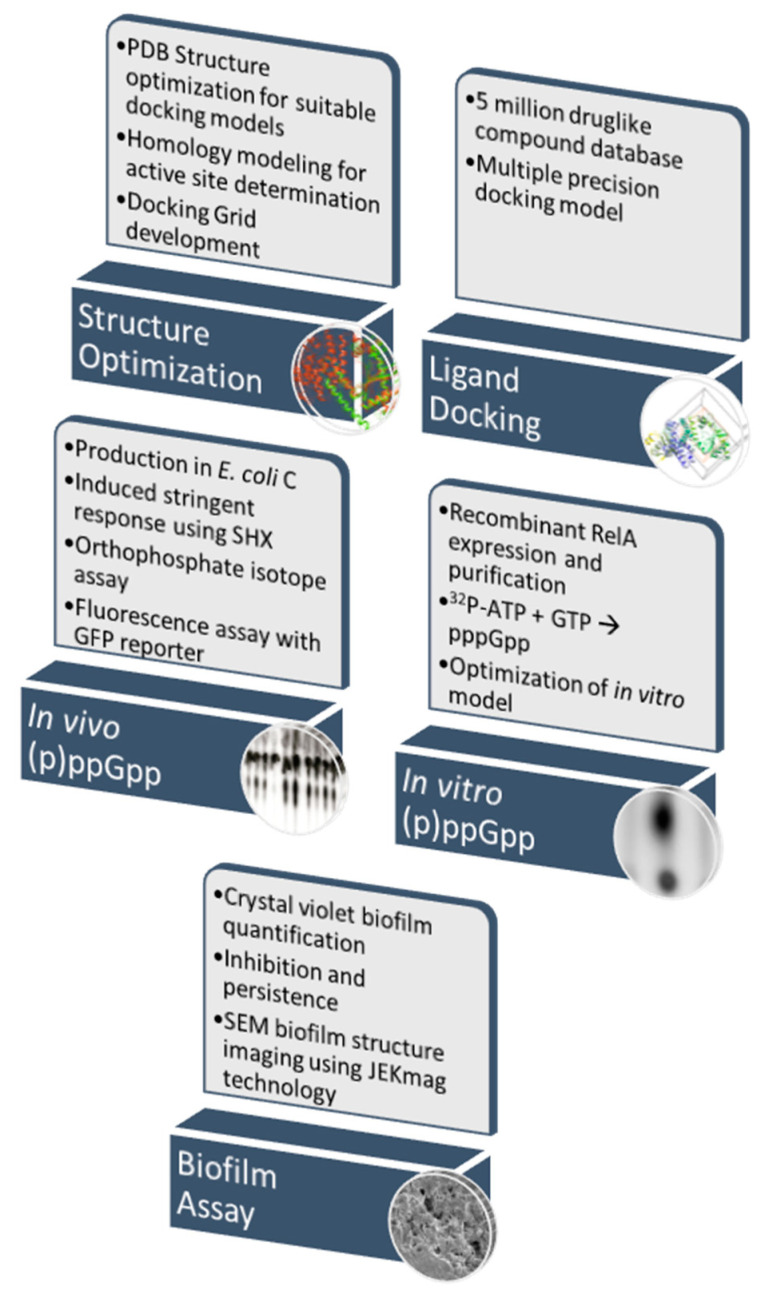
Schematic of pipeline for determination of effective RelA inhibitors.

Summarizing this work, structural modeling of the *E. coli* RelA protein [36] was performed to identify the active center. We then constructed multiple single amino acids substitution mutants of RelA based on this molecular modeling to confirm the location of the enzyme active center, and to confirm the critical role that the tyrosines Y310 and Y319 play in its enzymatic activity. Using the structural information gained from the *in silico* and laboratory studies, we then developed a computationally-based pipeline to identify RelA inhibitors from large databases of known compounds that provided for the screening of compounds in a relatively timely and cost-effective manner. Millions of compounds were screened in a matter of weeks and the ‘hit’ compounds were purchased for functional studies to determine their initial efficacy in laboratory-based in vivo and in vitro assays. The compound databases used for screening were designed to only include compounds that met the “drug-like” criteria for ligands, as defined by Lipinski’s rule of five [52]. This method has been shown to be highly effective in the discovery of drugs over the last 20 years and continues to improve in accuracy as the algorithms for ligand docking improve [53]. Using these *in silico* docking studies, two small-molecule compounds that were predicted to inhibit the RelA enzyme were identified. These compounds were then subjected to in vivo and in vitro (p)ppGpp quantification assays using the *E. coli* strain C and recombinant *E. coli* RelA enzyme, respectively, as well as in biofilm inhibition assays using our *E. coli* C biofilm model [50] (Figure 1).

### 3.1. Validation of the RelA Activity Assays

Several methods to study RelA enzymatic activity in vitro and in vivo have been published [25,26,28,48], and our methods were adapted from these sources. We performed two kinds of RelA activity tests: a ppGpp-dependent fluorescent reporter in vivo assay and direct (p)ppGpp detection assays in vivo and in vitro. The first method was based on the ability of ppGpp to affect expression of different genes [54]. One of these genes, *rpsJ*, encodes the 30S ribosomal protein S10 [55,56]. Its promoter, P*rpsJ*, belongs to the r-protein family of promoters, which is strongly inhibited by ppGpp and the DksA transcriptional factors [57,58]. Recently, a plasmid construct carrying a *yfp* (yellow fluorescent protein) gene driven by the P*rpsJ* was published [46]. This reporter plasmid contains the broad host range RK2 minimal replicon and is compatible with many other plasmid vectors. Comparison of the yellow fluorescent protein (YFP) activity between wild-type (WT) *E. coli* K12 and its *relA*^−^ mutant confirmed the effect of ppGpp production on P*rpsJ* activity and served as a validation of this method.

**Figure 2:**
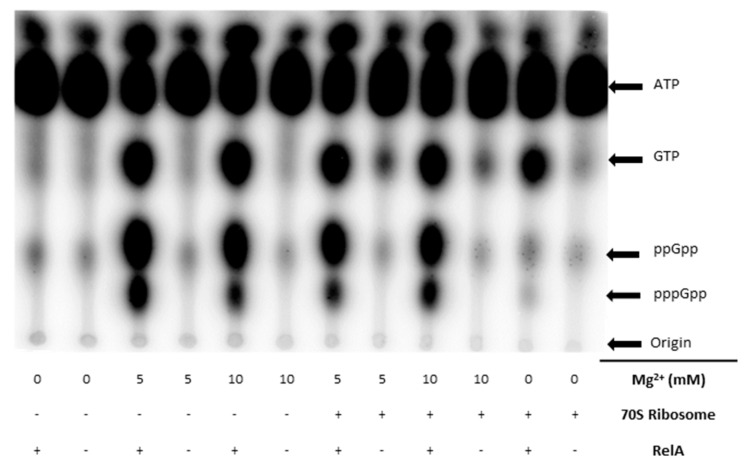
(p)ppGpp production assay. Qualitative production of (p)ppGpp in PBS buffer under various conditions: Mg^2+^ (10 mM and 5 mM), with and without 70S ribosome using γ-^32^P-ATP.

The direct (p)ppGpp detection in vivo and in vitro assays relied on different ^32^P radioactive nucleotides (γ-^32^P-ATP, α-^32^P-GTP) for use as substrates, and thin-layer chromatography (TLC) to separate the reaction products. Several methods were tested and optimized to give the best results for assessing the production of (p)ppGpp. It was found that the in vitro buffer system did not need to be phosphate free, as previously indicated [59]. It was also found that the concentration of magnesium needed to be above 5 mM for optimal synthesis of (p)ppGpp. Previous work has indicated that the 70S ribosome is needed for RelA to produce (p)ppGpp in vitro [60]; however, we found this not to be the case. There was no difference observed with 5 mM MgCl_2_ with and without 70S (Figure 2); therefore, it was not used in the in vitro reactions.

In the case of the in vitro assay, it was found that using γ-^32^P-ATP with 5 mM Mg^2+^ and no 70S ribosome was optimal to study the production of both ppGpp and pppGpp. In the case of the in vivo studies, [^32^P]-orthophosphate was used as the radiation source, and the cells then incorporated the ^32^P into (p)ppGpp. Both methods required TLC with a stationary phase of a polyethyleneimine (PEI)-cellulose plate and a mobile phase of 1 M potassium phosphate monobasic.

### 3.2. Homology Studies 

The active domain of the *E. coli* RelA cryo-EM (PDB: 5IQR) structure was determined using homology studies (Figure S1). Because there was no substrate bound to the RelA enzyme in the cryo-EM structure [36], we utilized two methods to determine the active site for molecular docking. The first method was a genomic-based homology method, where the known RelA protein sequences were compared, and the conserved residues were evaluated (Figure S1D). 

The second method was a structural homology method in which we used crystallographic data obtained from the *S. aureus* RSH-RelP that had been co-crystallized with its nucleotide substrates to identify both the pre- and post-catalytic active sites [37]. The alignment of the RelA and RelP predicted active site residues showed that they are structurally highly similar; this allowed for the identification and characterization of the active domain for targeting via ligand docking studies (Figure S1A). Using this information, we were able to determine two key amino acids involved in the binding of the first substrate in the catalytic process of GDP.

### 3.3. RelA Active Site Mutation Studies

To determine the accuracy of the *in silico* homology alignments and binding site determinations, two amino acid residues were identified as key to the catalytic activity of RelA, and then tested in the laboratory to ensure their assignment was correct (Figure S1). Tyrosine residues Y-324 and Y-332 (from the alignment) (Figure S1D) were determined to act as one of the largest contributors to the initial binding of GDP or GTP [37]. Y-324 was predicted to stabilize the phosphate of GDP/GTP by hydrogen bonding though means of its hydroxyl group, and Y-332 was predicted to be involved in π-stacking with the guanine’s aromatic ring. These stabilizations were predicted to allow for the initial binding of GDP/GTP within the active site. Y-324 and Y-332 residues correspond to the Y-310 and Y-319 residues of the *E. coli* RelA enzyme. We hypothesized that, if these residues were mutated to alanines (A-310 and A-319), this should bring about a decrease in the catalytic transfer of the pyrophosphate from ATP to form (p)ppGpp. Figure S2 shows the interactions of RelA with the native residues, as well as the lack of interactions when mutated to an alanine residue. To obtain the Y/A-310 and Y/A-319 substitutions of the *E. coli* RelA, we used two synthetic DNA cassettes to replace the 5′ end of the gene in the pJW2755-AM plasmid. The first *Psi*I/*Nsi*I (1144-bp) cassette contained a silent *Xba*I mutation [43,44] and the Y/A-310 substitution. The second 365-bp *Xba*I/*Nsi*I cassette introduced the Y/A-319 mutation (Figure S3). The 365-bp region between the *Xba*I (769) and *Nsi*I (1144) restriction sites contains the predicted RelA active center and can be easily exchanged with a synthetic construct to replace any of the tested amino acids.

**Figure 3:**
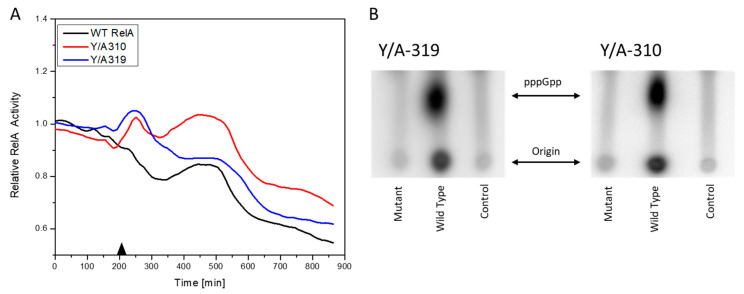
Effect of Y/A319 and Y/A310 substitutions on RelA enzymatic activity. (**A**) Ratio of relative RelA activity of the induced to non-induced cells in an in vivo fluorescence assay. The induction with 1.5 µM IPTG took place at 210 min, indicated with the black triangle. (**B**) In vitro pppGpp production assay. Control is [γ-^32^P] ATP without enzyme.

Two assays were conducted to evaluate the activity of the mutant RelA enzymes—an in vivo (p)ppGpp fluorescent reporter and in vitro (p)ppGpp production assay. The ASKA plasmid *p*JW2755-AM with the WT RelA protein and its Y/A-310 and Y/A319 versions were transformed into the *E. coli* AG1 strain containing a pAG001 plasmid, this plasmid contains a *yfp* gene expressed under a stringent response regulated promoter P*rpsJ* [46]. The *E. coli* AG1 strain contains a *relA1* mutation caused by an insertion of an *IS*2 insertion sequence between the 85^th^ and 86^th^ codons of the *relA* gene. These mutants retain a low level of (p)ppGpp synthesis activity [19]. Plasmids pJW2755AM and its derivatives, as well as pAG001, belong to different incompatibility groups and therefore can co-reside in a single cell. When plasmid encoded RelA expression is induced with isopropyl β-D-1-thiogalactopyranoside (IPTG), the cells produced (p)ppGpp. An increased level of (p)ppGpp decreased the level of YPF synthesis, as this was under the control of the P*rpsJ* promoter. The results showed a much higher reduction in YFP fluorescence in the case of the WT RelA protein than with its Y/A-310 and Y/A-319 derivatives (Figure 3A). In the in vitro assays, the purified proteins containing the Y/A-310 and Y/A-319, when compared with the WT protein, showed an even more striking reduction in pppGpp production (Figure 3B). These results confirm that the Y-310 and Y-319 amino acid residues play important roles in the enzymatic activity of RelA and, therefore, the active site, as modeled above, can be used as a target for the *in silico* docking of ligands for the identification of candidate druggable inhibitors.

### 3.4. In Silico Screening for Hit Compounds

Non-RelA components of the *E. coli* RelA cryo-EM (PDB: 5IQR) model including RNA and ribosome were stripped away from the file, leaving only the RelA structure (Figure S4). The RelA structure was then optimized using the Schrödinger Maestro protein preparation tools, including the package, Prime, which allows Maestro to fill in missing side chains and determine optimal amino acid orientations. The RelA enzyme was then structurally minimized using the force field OPLS3e [42] (Figure S4B). The enzyme binding pocket was determined using homology (Figure S2) studies, as well as a general understanding of RelA’s function, and a docking grid box was developed for protein ligand docking calculations.

Schrödinger Maestro Molecular Modeling Glide [61] was utilized to determine hit compounds, which were then validated using the laboratory assays described above to probe their ability to inhibit RelA activity. The Schrödinger Glide-HTVS mode was first used to screen the entire University of California, San Francisco Zinc^12^ Database of commercially available compounds. This database contains over 4 million compounds. The top 10% from the HTVS docking scan was then filtered into Glide-SP mode (standard precision). This output was then further refined and run in Glide-XP [61] mode (extra precision). These molecular docking studies resulted in two compounds showing a binding score that passed our threshold for binding affinity (Table 1) and were higher than those of the natural substrates ATP and GTP. These two compounds also fit both the Lipinski’s rule of five [52,62,63] for orally administered drugs, and the quantitative estimate of drug likeness [64,65].

### 3.5. Effect of S3-G1A and S3-G1B on (p)ppGpp Production via In Vitro and In Vivo RelA Assays

**Figure 4:**
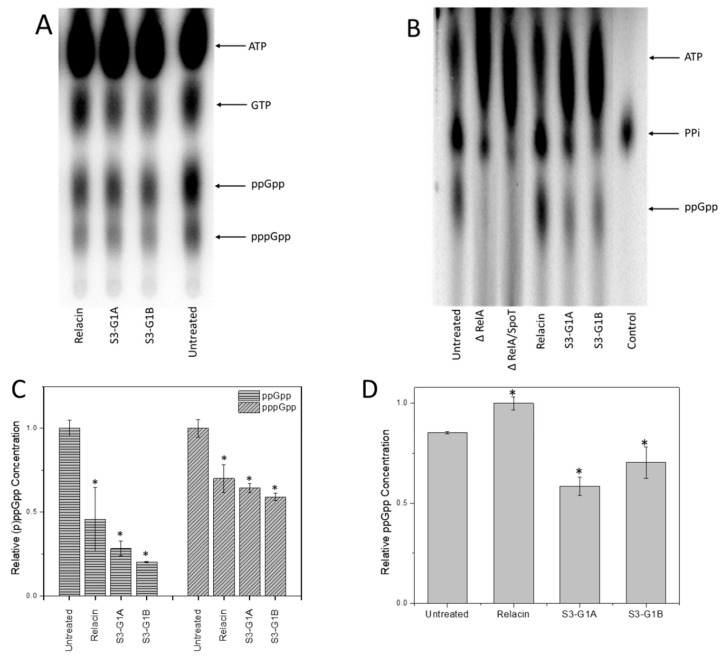
Effect of S3-G1A and S3-G1B compounds on RelA enzymatic activity. RelA (p)ppGpp production assay in vitro (**A**) and in vivo (**B**) treated with 20 µM of respective compound. Quantitative analyses of the in vitro (**C**) and in vivo (**D**) assays. * Indicates statistical significance.

After computational hit compounds were determined, the next step was to evaluate the effect of these small molecules on RelA activity in the in vitro and in vivo assays established above for the production of ppGpp. The results of the in vitro assay showed that both compounds S3-G1A (20 µM) and S3-G1B (20 µM) reduced ppGpp production when compared to an untreated sample by 71.7% (*p* < 0.0001) and 79.7% (*p* < 0001), respectively (Figure 4A,C). Both compounds showed higher reduction in activity than Relacin (20 µM) (45.4%, *p* = 0.0084). The in vivo assay showed a reduction in ppGpp production in samples treated with both compounds 31.4% (*p* = 0.0006) in S3-G1A and 17.75% (*p* = 0.0295) in S3-G1B. In this assay, no effect of Relacin on ppGpp production was observed (Figure 4B,D). We hypothesize that Relacin is not cell permeable and, therefore, does not influence in vivo ppGpp production. These results indicate that the S3-G1A and S3-G1B compounds are more efficient in vivo and in vitro than Relacin and validate the entire hybrid *in silico* laboratory pipeline. 

### 3.6. Effect of Hit Compounds on Bacterial Growth

Bacterial growth rates under conditions unrestricted by substrate availability are an indicator of cell health and viability. Despite great efforts to determine the role of the stringent response on the control of cell growth rate, general conclusions have not been able to be drawn [66,67,68]. However, all reports have shown that mutants unable to produce ppGpp grow slightly more slowly (up to 30%) than their cognate WT on all media tested [66,67,68]. We found that the initial growth rates for the WT strain and CF1652 (*relA*::Km) were the same (Figure S5). However, the growth of the WT strain started to slow down first after reaching OD_600_ = 0.6. The WT strain was expected to sense small changes in nutrient concentrations and react to it, reducing the growth rate. The *relA* mutant reached a higher cell density than that of the WT. After 18h of growth, both strains reached their highest cell densities and thereafter we observed varying decreases in OD_600_ values. We found that compounds S3-G1A and S3-G1B had no effect on planktonic growth rate. The maximal cell densities of the cultures with compounds were slightly lower than the control (Figure S6). 

### 3.7. Effect of Hit Compounds on Biofilm Inhibition and Dispersal

We have previously reported that *E. coli* strain C [50] is the only one of the five major “laboratory strains” of *E. coli* that is a superior biofilm former; therefore, this strain was used in our biofilm assays. Studies were conducted in 96-well high-throughput assays. In the biofilm inhibition assay, compounds were added to the wells at the beginning of the experiment. For the biofilm dispersal assay, the biofilm was allowed to grow for 24 and 48 h, the wells were washed with sterile phosphate-buffered saline (PBS), and fresh medium supplemented with the compounds was added to the wells. The amount of biofilm was measured after 24 h. There was no observed effect on the inhibition (Figure S7) or dispersal of biofilms with compounds alone.

### 3.8. Effect of Compound on Biofilm Persistence and Biofilm Viability

Biofilm persistence and viability were assessed with the hit compounds in combination with an antibiotic. It has been previously determined that sub-MICs of antibiotics result in increased biofilm formation [69]. Additionally, it has been previously demonstrated that the starvation response mediates high biofilm specific tolerance to antibiotics [70]. To check interactions between antibiotics and (p)ppGpp production, ampicillin was used in all of our assays due to its bactericidal effect and stressing effect. Sub-MIC concentrations of ampicillin were determined by growth measurements (OD_600_). We found that the biggest change in the culture cell density was observed between 40 and 60 µg/mL ampicillin (Figure S8A). Analyzing the effect of ampicillin on biofilm formation, we observed that the presence of the antibiotic significantly increased the amount of biofilm with the highest biomass observed at relatively high ampicillin concentrations (80 µg/mL) (Figure S8B). To analyze the effect of our hit compounds in combination with antibiotics, a range of ampicillin concentrations from 30 to 50 µg/mL was utilized.

**Figure 5:**
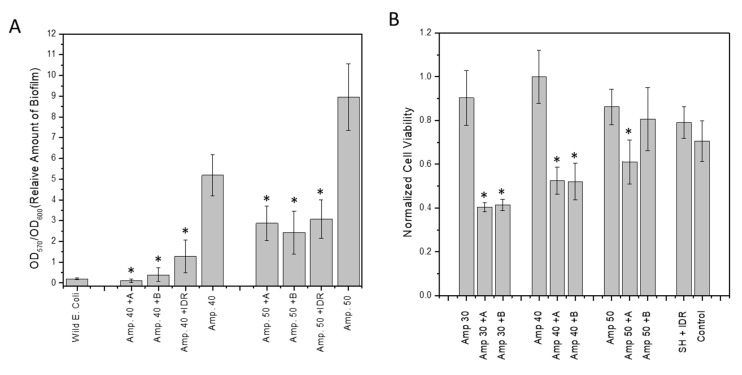
Synergistic effect of compounds and ampicillin on amount of biofilm and cell viability in biofilm. (**A**) Biofilm degradation utilizing hit compounds and ampicillin. A = S1-G1A (50 µM), B = S1-G1B (50 µM), Amp# = Ampicillin µg/mL, SH = serine hydroxamate, IDR = IDR 1018; (**B**) alamarBlue viability assay following combined treatment of cells with hit compounds and ampicillin showing a reduction in bacterial viability. * Indicates statistical significance.

The amount of biofilm biomass was determined in the combined presence of antibiotics and either compound A or B. These combination therapies led to a highly significant reduction in biofilm mass compared to the ampicillin-only treated controls (Figure 5A). As a reference control, we used IDR 1018, an antimicrobial peptide that is reported to target (p)ppGpp directly and degrade ppGpp in vitro [30]. The addition of the hit compounds to ampicillin concentrations of 40 µg/mL (Amp40) and 50 µg/mL (Amp50) resulted in a highly significant decreases in biofilm volume compared with their cognate antibiotic only treated control (Figure 5A). At Amp40 the biofilm biomass was reduced by 97.9% (*p* = 0.0009) for S3-G1A (50 µM), by 92.4% (*p* = 0.0014) for S3-G1B (50 µM), and by 75.4% (*p* = 0.006) for IDR1018 (6 µM). Amp50 showed reductions in biofilm biomass of 67.9% (*p* = 0.0044) for S3-G1A, of 72.9% (*p* = 0.0042) for S3-G1B, and 65.2% (*p* = 0.0054) for IDR1018. The difference between Amp40 and Amp50 can be attributed to the larger increase in biofilm volume induced by the higher concentration of antibiotic.

An alamarBlue cell viability assay also showed that ampicillin killed more bacterial cells when combined with the tested hit compounds (Figure 5B). In the case of ampicillin at 30 µg/mL (Amp30), the reductions were 55.4% (*p* = 0.0024) and 54.2% (*p* = 0.0027) for S3-G1A (50 µM) and S3-61B (50 µM), respectively. When higher concentrations of antibiotic were used, the synergetic effects of compounds S3-G1A and S3-G1B were less noticeable, with the decreases being only 29.2% (*p* = 0.0278) and 6.5% (*p* = 0.6), respectively. This effect was attributed to the greater volume of the biofilm contained in these samples (Figure 5B).

### 3.9. Effect of Hit Compounds on Biofilm Structure

**Figure 6:**
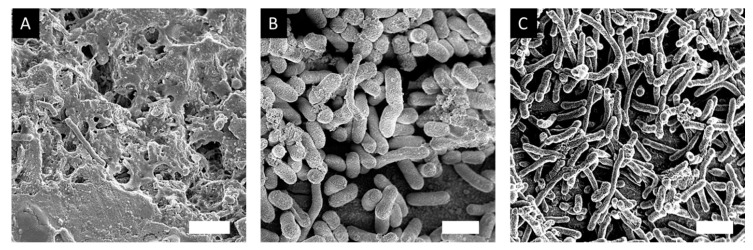
SEM images of *E. coli* C biofilm. (**A**) Untreated wild type *E. coli* C biofilm; (**B**) *E. coli* C biofilm treated with 40 µg/mL S3-G1A; C) *E. coli* C biofilm treated with 40 µg/mL S3-G1B. Scale bar = 1 µm.

Scanning electron microscopy (SEM) allowed us to probe the structure of the biofilms treated with the hit compounds. Biofilms were grown on metal pins for 3 days that were transferred daily to fresh LB medium using the JEKMag technique [51]. We found that, while there was not a large reduction in biofilm mass by the compounds alone, there was a very substantial change to the structure of the extracellular matrix of the biofilms. Biofilms treated with the ‘hit’ compounds (40 µg/mL S3-G1A and 40 µg/mL S3-G1B) exhibited a greatly reduced levels of matrix compared to untreated WT *E. coli* C (Figure 6). Treatment with S3-G1B also resulted in the elongation of the cells, indicating the possibility of an unknown off-target effect inducing filamentation.

## 4. Conclusions

We have established a hybrid *in silico* laboratory pipeline method to identify and characterize novel RelA inhibitors for the treatment of medically relevant bacterial biofilms in combination with traditional antibiotics. Using these reported methods in combination has given us the ability to identify and functionally characterize ‘hit’ compounds from a large database of *in silico* ligands. These methods have provided us with two lead compounds that are being utilized in downstream optimization structure–activity relationships to improve the efficacy of the core bio-isostere. The methods outlined here are important steps towards the process of finding an effective inhibitor of the RelA-driven bacterial stringent response and, in turn, the treatment of persistent biofilm infections. The computational components, which include binding site determinations and a multi-step docking process that incorporates a series of ever more stringent filters provides for the efficient screening of large ligand libraries, and provides an effective and cost-effective means for identifying hit molecules for the inhibition of RelA. Before the addition of these *in silico* methods, high-throughput ligand assays in the biofilm space were costly and time consuming.

## 5. Patents

United States Patent, Ji et al., Pub No. US 2020/0069647 A1, Pub. Data Mar. 4, 2020.

## Figures and Tables

**Table 1:** Hit compounds for the inhibition of RelA binding score. Binding score compared to the initial binding compound GTP.

Compound Structure	IUPAC Name	Short Name	Binding Score (kcal/mol)	Difference from GTP (kcal/mol)
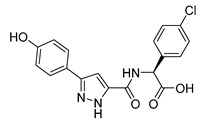	(4-chlorophenyl)([(3-(4-hydroxyphenyl)-1H-pyrazol-5-yl]carbonyl)amino)acetic acid	S3-G1A	−10.38	−1.71
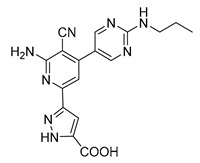	3-(6-amino-5-cyano-4-[2-(propylamino)pyrimidin-5-yl]pyridin-2-yl))-1H-pyrazole-5-carboxylic acid	S3-G1B	−9.64	−0.97
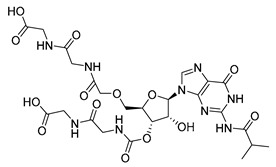	Relacin	R	−8.67	−0.08
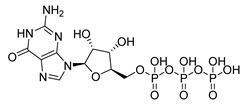	Guanosine triphosphate	GTP	−8.67	
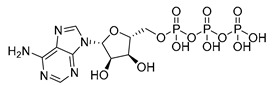	Adenosine triphosphate	ATP	−7.69	0.98

## Supplementary Materials

The following are available online at https://www.mdpi.com/2076-2607/8/9/1310/s1; Figure S1. RelA structure and sequence homology; Figure S2. RelA GDP interactions with and without amino acid mutations; Figure S3. RelA amino acid mutation scheme; Figure S4. Raw Cryo-EM structure of RelA (PDB: 5IQR); Figure S5. Effect of *relA* and *relA*/*spoT* mutations; Figure S6. Compound effect on planktonic growth of *E. coli* C; Figure S7. Effect of compounds on inhibition of *E. coli* C biofilm; Figure S8. Effect of ampicillin concentration on planktonic and biofilm growth; Table S1. Bacterial strains and plasmids utilized in this work.

## Materials and Correspondence

Correspondences should be address to Garth D. Ehrlich and Hai-Feng Ji. Materials and strain requests should be addressed to Jarosław E. Król.
