# Supplementary material for: The Development of a Pipeline for the Identification and Validation of Small-Molecule RelA Inhibitors for Use as Anti-Biofilm Drugs
Source: Microorganisms. 2020 Aug 28;8(9):1310. doi: 10.3390/microorganisms8091310 (PMC7563162; doi:10.3390/microorganisms8091310)
Supplement: Supplementary file 1 [file microorganisms-08-01310-s001.pdf]

SUPPLEMENTARY INFORMATION

The Development of a Pipeline for the Identification and Validation of Small-Molecule RelA Inhibitors for Use as Anti-biofilm Drugs

Donald C. Hall Jr[1,2], Jarosław E. Król[2], John P. Cahill[1], Hai-Feng Ji[1#], Garth D. Ehrlich[2,3#]

[1]*Department of Chemistry;* [2]*Center for Advanced Microbial Processing, Center for Genomic Sciences, Center for Surgical Infections and Bacterial Biofilms, Institute of Molecular Medicine and Infectious Disease; Department of Microbiology & Immunology;* [3]*Department of Otolaryngology-Head and Neck Surgery; Drexel University College of Medicine, Drexel University, Philadelphia, PA*

#: Corresponding Authors

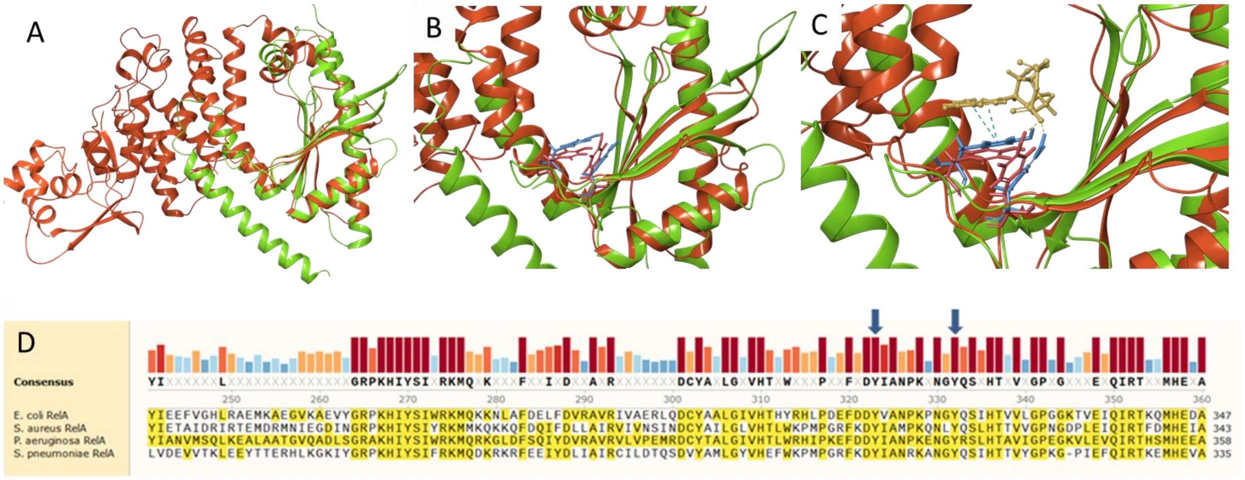

Figure S1. **RelA structure and sequence homology.** A) Structural alignment of known active site of *S. aureus* RelP (green) with *E. coli* RelA (orange); B) Alignment of *S. aureus* RelP (green) with *E. coli* RelA (orange) with two essential amino acids residues shown; C) GDP interactions with the essential residues can be seen with H-bonding and π-stacking shown in dashed lines; D) Protein sequence alignment of selected bacterial species (both Gram-positive and Gram-negative). Arrows denote conservation of the essential residues shown in B among most bacterial species shown

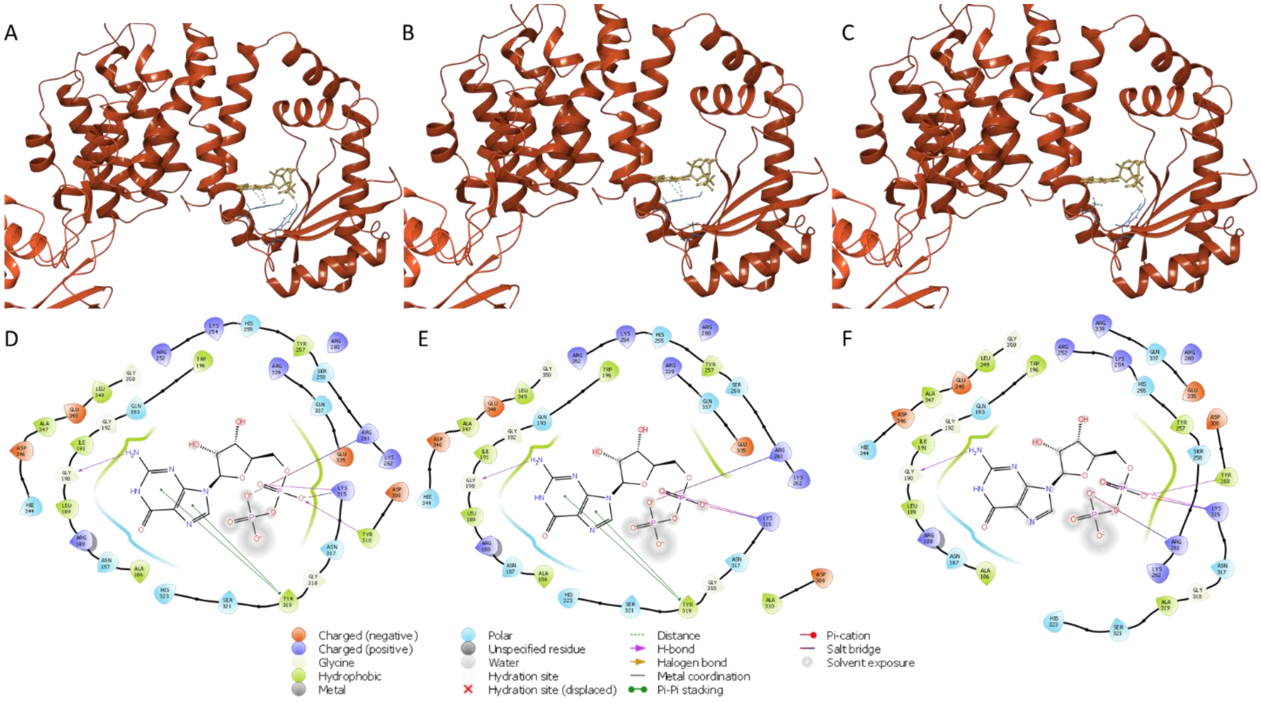

Figure S2. **RelA GDP interactions with and without amino acid mutations.** (A) 3D native RelA interaction (B) 3D Y/A-310 GDP interaction (C) 3D Y/A-319 GDP interaction (D) 2D native RelA interaction (E) 2D Y/A-310 GDP (F) 2D Y/A-319 GDP

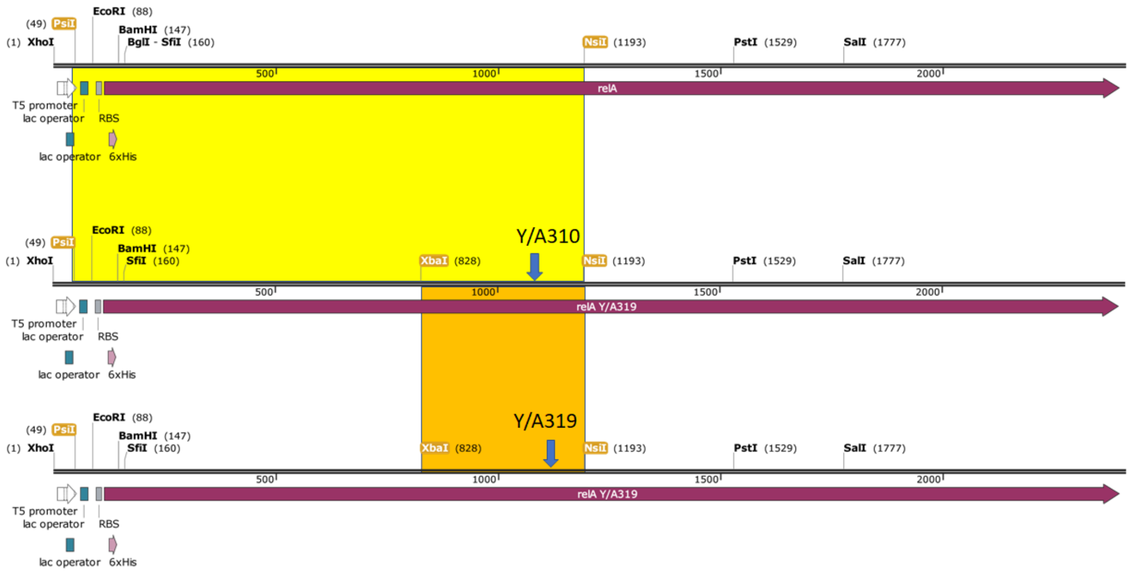

Figure S3. **RelA amino acid mutation scheme**. Arrows indicate where the mutation was introduced in the *relA* expression plasmid.

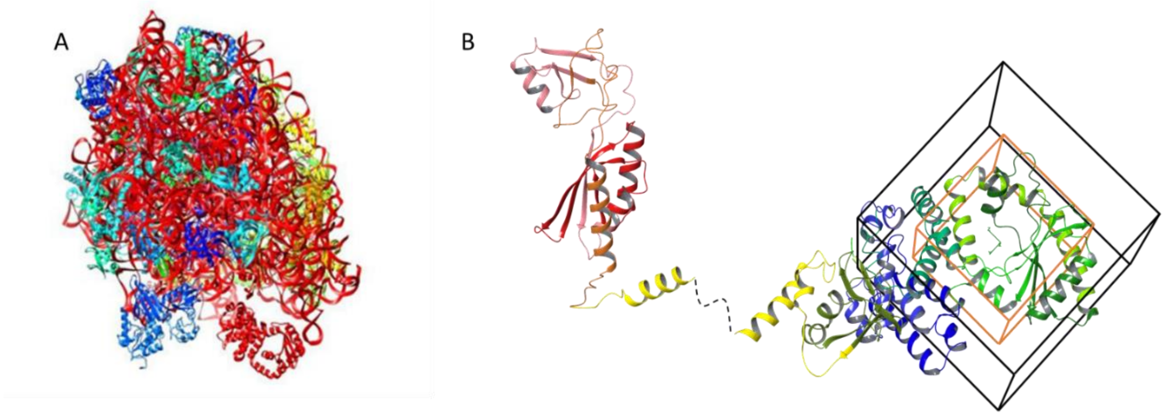

Figure S4. **Raw Cryo-EM structure of RelA (PDB: 5IQR).** RelA bound to a ribosome (A) and optimized RelA structure with docking grid box shown (B).

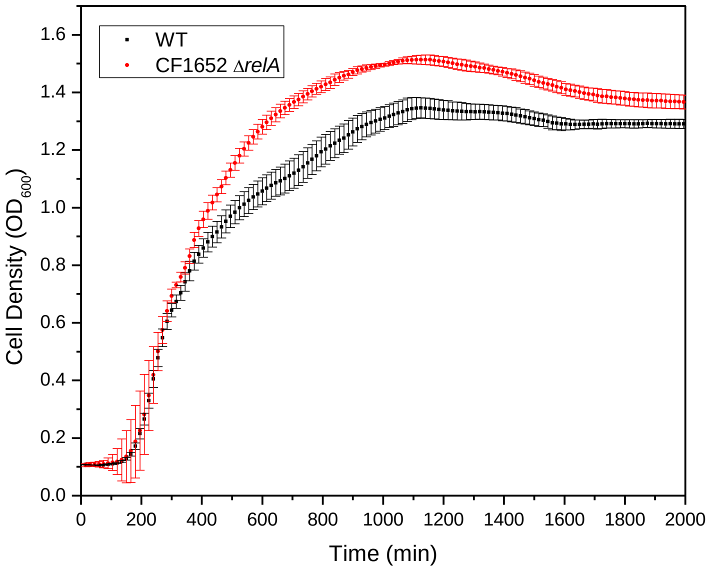

Fig S5. **Effect of *rel*A and *relA*/*spoT* mutations.** Overnight growth curves of Wild Type *E. coli* and the CF1652 Δ*relA* mutant.

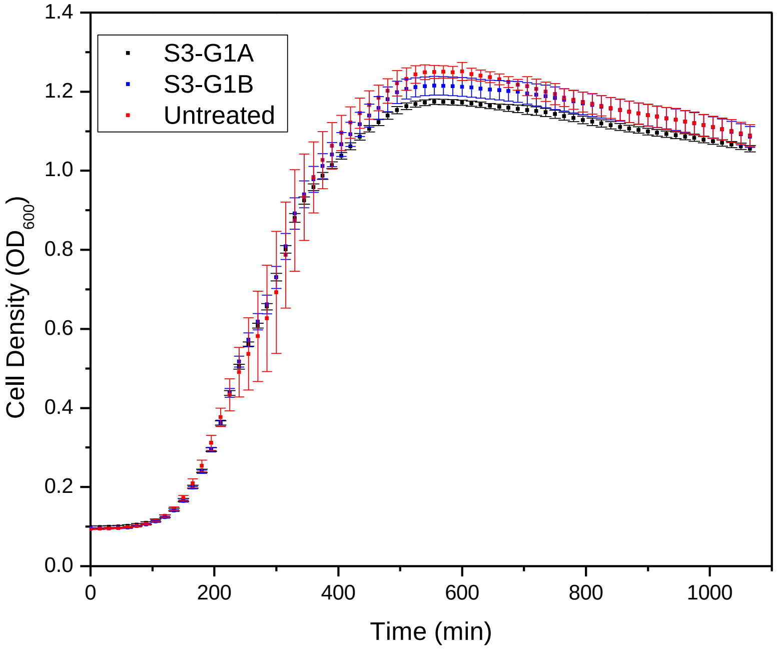

Figure S6. **Compound effect on planktonic growth of *E. coli* C.** *E. coli* C treated with compounds S3-G1A and S3-G1B (30 µg·mL^{-1}).

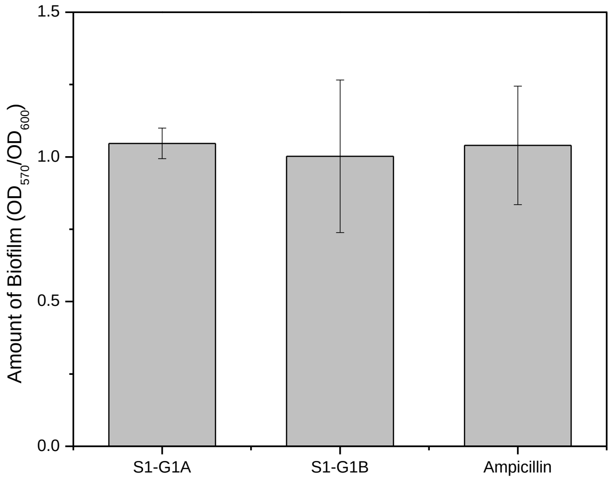

Figure S7. **Effect of compounds on inhibition of *E. coli* C biofilm.** S3-G1A and S3-G1B effect on the formation of biofilms using the crystal violet quantification method.

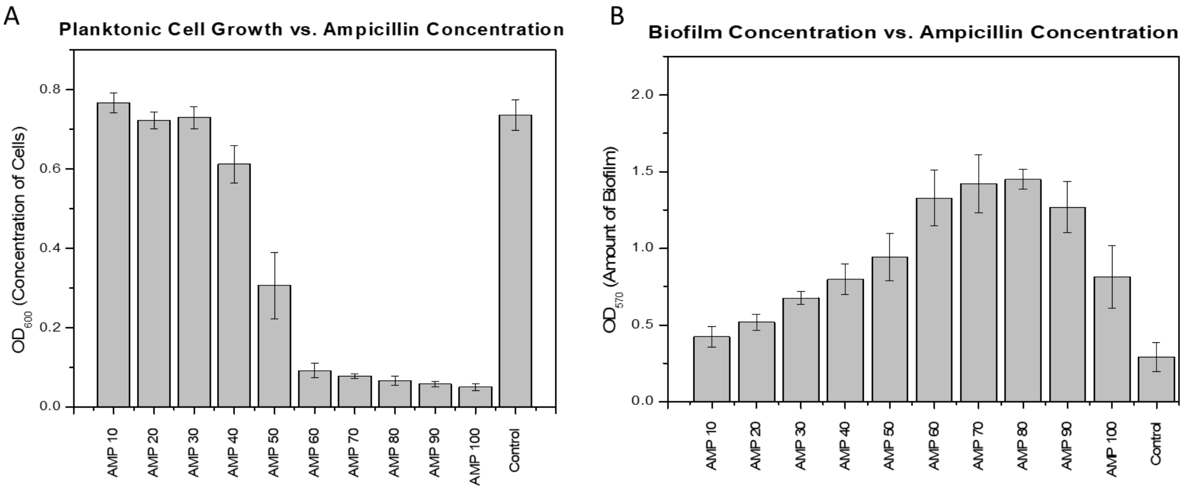

Figure S8. **Effect of ampicillin concentration on planktonic and biofilm growth**. A) Growth of cells in liquid culture at various concentrations of ampicillin (OD_{600}). B) Amount of biofilm formed at various concentrations of ampicillin. (AMP# = AMP at # µg·mL^{-1} concentration)

Table S1. **Bacterial strains and plasmids utilized in this work.** WT = wild type; Km = kanamycin; Cm = chloramphenicol

Strain and plasmid	Phenotype	Source
***E. coli* K12 MG1655**	WT	Lab collection
***E. coli* C**	WT	Lab collection
***E. coli* AG1 (ME5305)**	F^-,*recA*1 *endA*1 *gyrA*96 *thi*-1 *hsdR*17(r_k^- m_k^+) *supE*44 *relA*1	Kitagawa, M. *et al.*
***E. coli* CF1648**	WT	M. Cashel
***E. coli* CF1652**	*relA*::Km	M. Cashel
***E. coli* CF1693**	*relA*::Km, *spoT*::Cm	M. Cashel
**pJW2755-AM**	pCA24N(GFP-) with *relA* gene ASKA plasmid, Cm^R	Kitagawa, M. *et al.*
**pJEK2020-43**	pJW2755AM with *Xba*I silent site and Y/A319 mutation	This work
**pJEK2020-20**	pJW2755AM with *Xba*I silent site and Y/A310 mutation	This work
